# Race and Ethnicity and Sex Variation in COVID-19 Mortality Risks Among Adults Experiencing Homelessness in Los Angeles County, California

**DOI:** 10.1001/jamanetworkopen.2022.45263

**Published:** 2022-12-06

**Authors:** Natalie A. C. Porter, Hannah K. Brosnan, Alicia H. Chang, Benjamin F. Henwood, Randall Kuhn

**Affiliations:** 1 Department of Community Health Sciences, Fielding School of Public Health, University of California, Los Angeles; 2 Los Angeles Department of Public Health, Los Angeles, California; 3 Dworak-Peck School of Social Work, University of Southern California, Los Angeles

## Abstract

**Importance:**

Few studies have used precise age-specific data to construct age-standardized estimates of the relative risks (RRs) of COVID-19 mortality for people experiencing homelessness (PEH) vs the general population, and none to date has addressed race and ethnicity and sex variations in COVID-19 mortality among PEH with COVID-19 infection.

**Objective:**

To measure age-standardized mortality rate ratios for PEH vs the general population overall and by sex and race and ethnicity.

**Design, Setting, and Participants:**

In this cross-sectional study, crude and age-specific COVID-19 mortality rates per 100 000 people were calculated using 5-year age groups and standardized mortality ratios for PEH and the general population aged 25 years and older, assessing differences by race and ethnicity and sex, from January 1, 2020, to November 1, 2021. Mortality and population estimates came from COVID-19 mandatory case reporting conducted by the Los Angeles County Department of Public Health, the annual point-in-time homeless count, and the US Census.

**Main Outcomes and Measures:**

The main outcome was COVID-19 deaths sourced from clinician reports, death certificates, medical examiner reports, and vital records deaths. PEH status was determined using the US Department of Housing and Urban Development definitions for homelessness at the time of COVID-19 diagnosis or symptom onset.

**Results:**

The study population included 25 441 deaths among an estimated 6 382 402 general population individuals and 256 deaths among an estimated 52 015 PEH. The race and ethnicity of the PEH sample was as follows: 15 539 Black (29.9%), 18 057 Hispanic (34.7%), 14 871 female (28.6%), 37 007 male (71.3%), and 3380 aged 65 years or older (6.5%), compared with the estimated general population of 6 382 402, which was 591 003 Black (9.3%), 2 854 842 Hispanic (44.7%), 3 329 765 female (52.2%), 3 052 637 male (47.8%), and 1 190 979 aged 65 years or older (18.7%). Crude death rates were 0.49% for PEH and 0.40% for the general population, but PEH experienced age-specific COVID-19 mortality risk 2.35 (95% CI, 2.08-2.66) times higher than the general population. There was significant risk associated with PEH status compared with their counterparts in the general population for Black PEH (RR, 1.69; 95% CI, 1.31-2.18), Hispanic PEH (RR, 2.34; 95% CI, 1.96-2.79), White PEH (RR, 8.33; 95% CI, 6.37-10.88), female PEH (RR, 3.39; 95% CI, 2.56-4.48), and male PEH (RR, 1.74; 95% CI, 1.52-2.00).

**Conclusions and Relevance:**

This cross-sectional study of COVID-19 mortality among PEH with COVID-19 infection provides evidence suggesting excess risk of age-adjusted COVID-19 mortality among PEH compared with the general population. This study furthers understanding of the intersectional association between homelessness and race and ethnicity, as higher levels of mortality but narrower racial disparities among PEH than in the general population were observed.

## Key Points

**Question:**

Did COVID-19 mortality differ between people experiencing homelessness (PEH) in Los Angeles and the general population by race and ethnicity and sex?

**Findings:**

In this cross-sectional study, PEH with COVID-19 infection experienced a higher risk of COVID-19 mortality than the general population among all racial and ethnic and sex groups. White PEH had greater relative risk of mortality vs their general population counterparts than Black and Hispanic PEH. Female PEH had greater relative risk than male PEH.

**Meaning:**

The findings of this study provide evidence suggesting excess risk of COVID-19 fatality among PEH with COVID-19 infection and further our understanding of the intersectional association between homelessness and race and ethnicity and sex.

## Teaser

This cross-sectional study examines variation by race, ethnicity, and sex of mortality rates of people experiencing homelessness who develop COVID-19 compared with the general population.

## Introduction

The burden of homelessness falls disproportionately on health disparity populations.^1,2,3,4,5^ Homelessness exacerbates health disparities and results in the early onset of geriatric conditions,^6,7,8^ contributing up to 2 decades of decreased life expectancy for people experiencing homelessness (PEH) compared with the general population.^9,10,11,12,13,14^ Concerns for such accelerated aging led to successful calls for federal funding to protect PEH from COVID-19 exposure by placing PEH into protective housing programs, reducing shelter density, conducting COVID-19 case detection among PEH, and increasing vaccination priority for PEH.^15,16^ The hope was that these interventions would result in COVID-19 having no worse outcomes in PEH than the general population despite their existing vulnerabilities. Using data from Los Angeles (LA) County, California, this study measured relative risks (RRs) of COVID-19 mortality for PEH compared with the general population among those with COVID-19 infection and looked at variations by race and ethnicity and sex to examine whether local interventions were successful at preventing COVID-19 mortality among PEH.

Our study takes an intersectional perspective that considers the powerful role of racial and ethnic disparities in both COVID-19 mortality and the experience of homelessness.^17^ Observational studies and systematic reviews have consistently documented the excess risk of COVID-19 mortality among Black or African American (hereafter, Black) and Hispanic or Latino (hereafter, Hispanic) populations compared with non-Hispanic White populations, with one study^18^ suggesting excess age-adjusted mortality of 80% and place-adjusted mortality of 50%. A recent study^19^ in LA County found that Hispanic individuals experienced a 3 times higher age-adjusted mortality risk due to COVID-19 than White individuals, sufficient to reverse the long-standing Hispanic mortality advantage in the county. In the US, Black individuals account for 13% of the general population but 40% of the homeless population nationally, and 8% of the LA County population but an estimated 33% to 40% of LA County PEH.^1,20^ Although Hispanic individuals make up slightly less of LA County’s homeless population than the general population, their rates of homelessness are increasing, and evidence suggests unsheltered homeless Hispanic individuals experience unique socioeconomic and behavioral risk profiles.^21,22^

We also considered intersections with sex or gender. It has been well documented that women have experienced lower age-specific COVID-19 mortality risks than men due in large part to the presence of fewer aggravating comorbidities. Women are underrepresented among PEH, constituting 30% to 40% of the population.^23^ However, women experiencing homelessness, particularly those who are unsheltered, may also bear a higher burden of socioeconomic risk factors than men experiencing homelessness.^14,24^

To date, several studies^25,26,27,28,29^ have offered clues that PEH have experienced elevated age-specific mortality risks due to COVID-19. However, these studies were limited by the lack of publicly available disaggregated data on PEH with COVID-19 infection, leading to their inability to fully examine the intersections of sex and race and ethnicity with PEH status in studying COVID-19 mortality. As of December 2021, available data^27,30,31,32^ suggested that approximately 30% of COVID-19 tests among PEH were positive—higher than the 22% positive test rate for all tests reported to the Centers for Disease Control and Prevention. Studies of COVID-19 affecting PEH in France, Belgium, and the US have found higher rates of infection and mortality due to COVID-19 relative to the general population.^26,27,28,33^ This higher infection and mortality risk among PEH is likely due to a combination of factors, including accelerated senescence, covulnerability of opioid use, incarceration, congregate shelter environments, and limited ability to engage in protective behaviors such as social distancing.^34,35,36,37,38^ However, a recent study^29^ of deaths among PEH in San Francisco from March 17, 2020, to March 16, 2021, observed very few decedents who had been diagnosed with COVID-19. None of these studies has addressed the intersections between PEH status and racial and ethnic or sex disparities in COVID-19.

The current study was situated in LA County, which is home to 10% of the US PEH population and the largest unsheltered population. In the years leading up to the pandemic, there has been a widespread and increasing mortality crisis among LA County’s homeless population due to a broad spectrum of environmental and behavioral causes.^39^ To examine the RRs of COVID-19 mortality among PEH in LA County, this study linked comprehensive COVID-19 mortality data from LA County’s active surveillance systems to age, sex, and race and ethnicity distribution data drawn from a large-scale survey conducted as part of a federally mandated count of homeless individuals. Our objectives were to measure the pattern and extent of age-specific acceleration of COVID-19 risk among PEH and measure age-standardized mortality rates (ASMRs) for PEH vs the general population overall and by sex and race and ethnicity. We expected that PEH with COVID-19 infection would experience greater mortality due to COVID-19 and that we would observe disparities by race and ethnicity and sex.

## Methods

The UCLA (University of California, Los Angeles) Institutional Review Board stated approval was not needed for this cross-sectional study because only aggregate data were used. This study followed the Strengthening the Reporting of Observational Studies in Epidemiology (STROBE) reporting guideline for cross-sectional studies.

The LA County Department of Public Health routinely investigated COVID-19 deaths for indications of homelessness to surveil disease incidence in this population. PEH included those who met the US Department of Housing and Urban Development definitions for homelessness, including those who sleep in emergency shelters and in unsheltered settings, such as on the street, in tents and vehicles, or in groups at encampments.^40^ PEH status, race and ethnicity, and sex were coded based on case interviews, database matches against the Homeless Management Information System, provider reports, medical examiner reports, shelter operator or social worker reports, and family member interviews when the case was not available. Confirmed COVID-19–related deaths were defined in accordance with guidance from the Council of State and Territorial Epidemiologists and the California Department of Public Health. All traumatic and accidental deaths, defined as a sudden and/or unexpected death (eg, through violence, accident, drug overdose, or unforeseen medical event) and not due to illness, were excluded. Confirmed COVID-19 deaths included those with a positive SARS-CoV-2 molecular test and either COVID-19 listed as a cause of death on the death certificate or the date of death occurring within 60 days of the first confirmed positive molecular test (up to 90 days if the patient was intubated). This method is consistent with epidemiologic surveillance and helps to account for the lower use of health care among PEH. Individuals with COVID-19–related death were categorized as PEH when they were determined to have been PEH at the time of diagnosis or death. Our estimates reflect deaths occurring from January 1, 2020, through November 1, 2021.

Population estimates for PEH were based on the January 2020 point-in-time count of homeless individuals for the LA County Continuum of Care in accordance with the method used for that count. For LA County, the Continuum of Care includes all of LA County except Glendale, Long Beach, and Pasadena.^41^ Age, sex, and race and ethnicity distribution estimates came from a postenumeration survey conducted from January to March 2020, in which 4304 current PEH were interviewed using a stratified random sample. Because age and race and ethnicity composition estimates were based on a sample survey, we conducted sensitivity analysis that extended estimated CIs to account for the effect of uncertainty in the population denominators. LA County general population estimates were derived from US Census Population Estimates (with Pasadena, Glendale, and Long Beach removed).^42^ We used the sum of the LA County general plus PEH population as the reference population for all age standardizations.

### Statistical Analysis

We calculated crude and age-specific COVID-19 mortality rates per 100 000 population and ASMRs with SEs and 95% CIs for PEH and the general population aged 25 years and older.^43^ Younger individuals were omitted due to their lower susceptibility to COVID-19 mortality. Age-specific rates were also obtained for male and female PEH and LA County general population, and for Black, Hispanic, and White subgroups. To test for between-group differences in ASMR and age-specific mortality, we estimated mortality rate ratios (MRRs) using a post hoc Poisson regression with age-specific deaths as the dependent variable, age-specific population size as the exposure term, controls for 5-year age category, and controls for PEH status, race and ethnicity, and sex. We report MRR estimates and 95% CIs.

In addition, we conducted sensitivity analysis to test the use of alternative population standards by replacing the central estimate of the PEH population in each age, sex, and race and ethnicity group with a population that was 2 SEs higher, thus accounting for the possibility that our results were affected by an underestimate of PEH population in a particular group. All calculations were performed in Excel, version 16.57 (Microsoft Corp) and Stata, version 16.1 (StataCorp LLC).

## Results

The study included 52 015 PEH, of whom 29.9% (n = 15 539) were Black, 34.7% (n = 18 057) were Hispanic, 28.6% (n = 14 871) were female, 71.3% (n = 37 007) were male, and 6.5% (n = 3380) were aged 65 years or older. Between March 1, 2020, and November 1, 2021, the data captured 256 deaths among PEH with COVID-19 infection, for a 0.49% crude death rate (256/52 015). This finding included 50 deaths among women vs 205 among men (1 decedent did not fit either category) and the breakdown of PEH deaths by race and ethnicity was as follows: 62 Black, 122 Hispanic, and 55 White. Due to low numbers of the racial and ethnic category other, we could not disaggregate from within this category to draw meaningful conclusions or compare with general population estimates. The study included 189 deaths occurring the first year of the pandemic through March 2021, with most occurring during the Delta variant surge of December 2020 through February 2021.^33^ An additional 67 deaths occurred from March 1 to November 1, 2021. We compared these PEH with an estimated 6 382 402 individual adults in the general population, of whom 9.3% (591 003) were Black, 44.7% (2 854 842) were Hispanic, 52.2% (3 329 765) were female, 3 052 637 (47.8%) were male, and 18.7% (1 190 979) were aged 65 years or older. We observed 25 441 deaths among the general population with COVID-19 infection for a 0.40% crude death rate (25 441/6 382 402).

### COVID-19 Mortality Among PEH and the General Population

**Figure 1.**
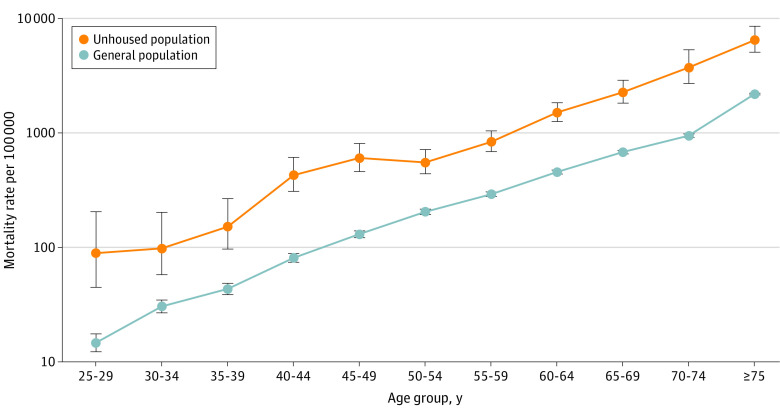
COVID-19 Age-Specific Mortality Rates in People Experiencing Homelessness vs General Population, 5-Year Age Groups Error bars represent 95% CIs.

The Table reports ASMRs and MRRs. PEH with COVID-19 infection experienced a 2.35 (95% CI, 2.08-2.66) times higher age-adjusted risk of COVID-19–associated mortality than the general population, with an ASMR of 880 (95% CI, 715-1046) per 100 000 among PEH and 392 (95% CI, 387-396) per 100 000 among the general population (Table). Figure 1 shows that absolute rates of COVID-19 mortality increased with age among both PEH and the general population. We observed a more pronounced relative mortality risk among PEH at younger ages, with PEH between the ages of 35 and 44 years experiencing 3.65 (95% CI, 3.27-4.15) times the COVID-19 mortality of their general population counterparts (Figure 1). Increased risks for PEH persisted at all ages, but the risk ratio tended toward parity at older ages, with adults aged 75 years or older having MRR of 1.70 (95% CI, 1.05-1.89).

**Table.** Age-Standardized Mortality Rate per 100 000, 5-Year Age Groups^a^

Demographic	Rate (95% CI), per 100 000		PEH vs general population, from Poisson regression, MRR (95% CI)
	Homeless population	General population	
Total	880 (715-1046)	392 (387-396)	2.35 (2.08-2.66)
Black	597 (393-801)	378 (362-393)	1.69 (1.31-2.18)
Hispanic	1450 (1063-1837)	625 (615-636)	2.34 (1.96-2.79)
White	612 (355-870)	133 (130-137)	8.33 (6.37-10.88)
Black vs White, MRR (95% CI)	0.94 (0.66-1.36)	2.84 (2.70-2.97)	NA
Hispanic vs White, MRR (95% CI)	2.25 (1.63-3.10)	4.83 (4.69-4.98)	NA
Female	1102 (453-1751)	285 (279-290)	3.39 (2.56-4.48)
Male	871 (700-1043)	523 (515-532)	1.74 (1.52-2.00)
Male vs female, MRR (95% CI)	1.20 (0.88-1.65)	1.84 (1.79-1.88)	NA

Abbreviations: MRR, mortality rate ratio; NA, not applicable.

^a^ Age groups were top-coded at 75 years, meaning that all counts above age 75 years were collapsed into 1 category (eg, 75-79, 80-84, and ≥85 years).

### COVID-19 Mortality Among PEH by Race and Ethnicity

While all PEH experienced greater mortality risk due to COVID-19 relative to the general population, considerable racial and ethnic disparities existed in both groups. Within the PEH population, Hispanic individuals experienced the highest ASMR at 1450 (95% CI, 1063-1837) deaths per 100 000. Hispanic PEH had a 2.25 (95% CI, 1.63-3.10) higher risk of mortality due to COVID-19 than White PEH—a ratio much lower than in the general population in which Hispanic individuals had 4.83 (95% CI, 4.69-4.98) times greater mortality risk. Mortality for Black PEH did not differ significantly from that of White PEH (MRR, 0.94; 95% CI, 0.66-1.36), in contrast to the significantly higher Black vs White mortality risk in the general population (MRR, 2.84; 95% CI, 2.70-2.97).

### COVID-19 Mortality Within Racial and Ethnic Groups by PEH Status

**Figure 2.**
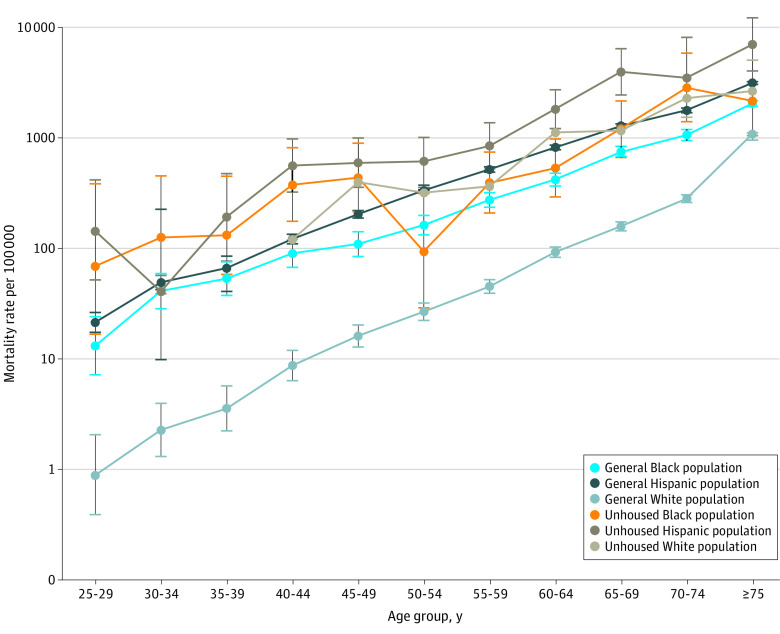
COVID-19 Age-Specific Mortality Rates in People Experiencing Homelessness vs General Population, 5-Year Age Groups, by Race Error bars represent 95% CIs.

White PEH experienced the greatest within-group MRR, with 8.33 (95% CI, 6.37-10.88) times higher risk of COVID-19 mortality than the White general population. This outcome appears to be associated with the low mortality rate among White people in the general population who, compared with other racial and ethnic groups, experienced lower mortality rates at all ages (Figure 2). Despite the high mortality for Hispanic and Black individuals in the general population, we observed significant RR of COVID-19 mortality associated with PEH status, with Hispanic PEH having 2.34 (95% CI, 1.96-2.79) times higher risk than the general Hispanic population and Black PEH having 1.69 (95% CI, 1.31-2.18) times higher risk than the Black general population.

### Sex Differences in COVID-19 Mortality

**Figure 3.**
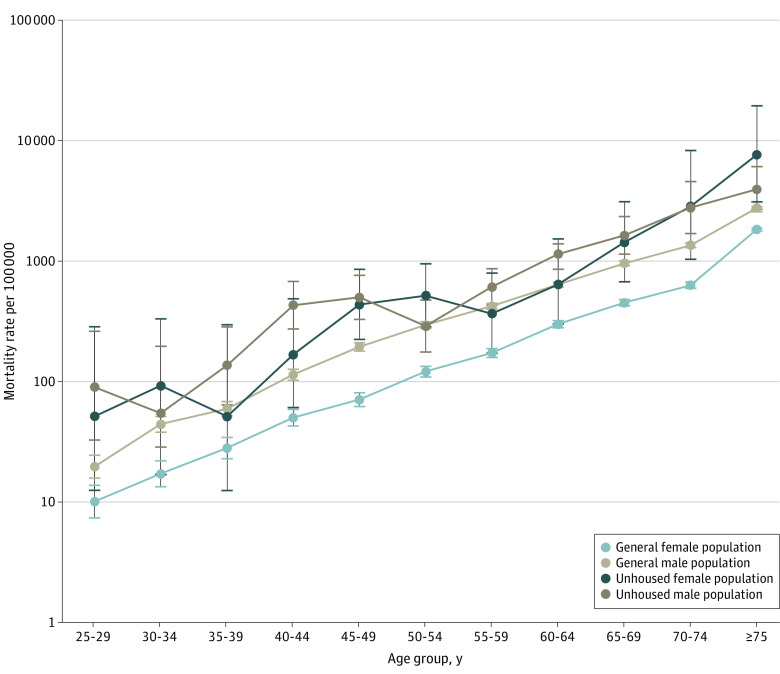
COVID-19 Age-Specific Mortality Rates in People Experiencing Homelessness vs General Population, 5-Year Age Groups, by Sex Error bars represent 95% CIs.

Although male individuals in the general population experienced higher mortality risk due to COVID-19 compared with female individuals (MRR, 1.84; 95% CI, 1.79-1.88), this association was diminished among PEH. Male PEH experienced similar mortality risk to female PEH (MRR, 1.20; 95% CI, 0.88-1.65). Looking within sex, female PEH experienced an MRR 3.39 (95% CI, 2.56-4.48) times greater risk of COVID-19 mortality compared with female individuals in the general population, while male PEH experienced an MRR 1.74 (95% CI, 1.52-2.00) times greater risk than those in the general population (Figure 3).

To test the robustness of these results to uncertainty in the estimated age-, sex-, and race and ethnicity–specific denominators for the homeless population, we reran our analyses replacing the estimated mean population denominators for PEH with the estimated 95% CI value for the denominator, which had little effect on the final results. While these changes altered the significance of some individual 5-year estimates, they did not affect the significance of any differences in PEH-general risk ratios for age, race and ethnicity, or sex.

## Discussion

These new estimates from LA County document the excess COVID-19 mortality experienced by PEH with COVID-19 infection relative to the general COVID-19–infected population occurring at all ages, with higher RR occurring earlier in the life course. Across most ages, our results are highly consistent with a 10-year acceleration of COVID-19 mortality risk among PEH. Taken together, these standardized rates reveal that PEH were 2.4 times more likely to die while infected with COVID-19 compared with the general population. Our findings are consistent with studies^1,18,19^ highlighting accelerated senescent decline among PEH. These findings suggest both that homelessness is a unique risk factor for COVID-19 and that the likely mechanism is through vulnerability brought on by accelerated aging. Our findings are also consistent with estimates drawn from most other jurisdictions.^25^ We note that one recent study^44^ in San Francisco County observed no PEH deaths attributed to COVID-19 and just 4 decedents who tested positive. Although the much smaller population size and COVID-19 mortality burden in San Francisco County vs LA County make it difficult to compare these results statistically, that study offers evidence that it was possible to avert excess COVID-19 mortality through shelter dedensification, protective hotel programs, and contact tracing and that some portion of PEH deaths in LA County were preventable.

We observed increased risks for PEH with COVID-19 infection across all race and ethnicity and sex groups, but the combination of PEH status and high-risk race and ethnicity or sex group did not have a multiplicative outcome. The age-specific race and ethnicity patterns shown in Figure 2 note that the White general population experienced better outcomes compared with all other groups, both housed and unhoused. White PEH experienced an 8-fold increase in COVID-19 mortality risk compared with the White general population and experienced age-specific patterns consistent with a 15-year acceleration of age-specific risk. PEH status risks were less pronounced for Black and Hispanic populations, and thus race and ethnic disparities were far less pronounced among PEH than in the general LA County population or the broader US.^18^ This pattern of compressed racial and ethnic disparities may reflect a ceiling effect on the maximum level of COVID-19 risk that can be observed even in the presence of multiple negative competing risks. However, it may also reflect differences in the composition and experiences of White vs Black and Hispanic PEH. A growing number of studies suggest that entry into homelessness for Black individuals in the US may be influenced primarily by structural factors, while homelessness among White individuals in the US is more likely influenced by individual factors, such as psychiatric conditions and substance use.^45^ White PEH may thus be more likely to experience more intense weathering and early onset of age-related conditions.^24,46^ We note that this finding is also consistent with recent studies of the intersectional effects of adverse circumstances and race and ethnicity on allostatic load, which have found that advantages for White individuals are less pronounced among disadvantaged than advantaged groups.^47,48^

The pattern of excess mortality risk among Hispanic PEH is striking, both relative to the general Hispanic population and to other PEH. The ASMR for Hispanic PEH with COVID-19 infection (1450 per 100 000) was 11 times higher than for the White general population (133 per 100 000). High COVID-19 mortality rates among Hispanic individuals are consistent with statewide trends showing excess deaths among the Hispanic population, especially for those of working age, Mexican or Central American descent, without a high school degree, or working in industries including food, agriculture, and manufacturing.^49^ Unsheltered Hispanic PEH are more likely to have lower rates of public benefit use and higher rates of employment compared with unsheltered Black and White PEH, indicating that Hispanic PEH may experience similar risk factors to housed Hispanic individuals but with heightened vulnerability.^21^ This effect could also result from more rapid growth in the Hispanic PEH population than in other groups, perhaps due to pandemic-related disruptions to housing markets.^50^

Relative risks among PEH with COVID-19 infection were higher than the general population for both male and female PEH but were substantially higher for women. As with race and ethnicity, this pattern could again be consistent with a competing risks framework or with a model of negative selection into homelessness and more substantial adverse experiences among women.^51,52^ The increased risk of COVID-19 mortality for female PEH echoes persistent concerns about the well-being of women experiencing homelessness in LA County and beyond and could also reflect differential access to care among female vs male PEH.^53,54^ This result is consistent with a meta-analysis^14^ that found higher rates of all-cause mortality among disadvantaged female individuals compared with male individuals.

### Limitations

This study has limitations. First, these results only report on mortality and do not address infection or hospitalization rates. Although such data are available, more in-depth efforts are needed to account for known biases in access to testing (especially asymptomatic testing) and hospital facilities. Given successful efforts to place high-risk PEH in hotels and other temporary housing throughout much of the pandemic, PEH may have had lower rates of infection than the general population and thus our findings may represent a lower bound of the true physiologic vulnerability facing PEH.^55^ Second, we used aggregate subgroup data and thus cannot account for the specific comorbidities, confounders, or variations in access to care that might explain our results. There is a need for studies that link electronic health record data on comorbidities to homelessness status and subsequent COVID-19 outcomes, although such studies are rare and often limited to specific health networks. A more immediately feasible goal would be an analysis of differences by time period that could potentially assess whether lower rates of vaccine uptake among PEH resulted in widening mortality differentials over time.^56^ Third, our analysis used denominators based on the population in 2020 and thus did not account for potential changes in the size or composition of the general or PEH populations over time.^33^ We note, however, that our results hold if we restrict the findings to deaths occurring through March 2021. Insufficient counts limited our ability to include other racial and ethnic groups and genders (ie, transgender, Asian PEH) or to test 3-way intersections between race and ethnicity, sex, and PEH status. In addition, LA County, like many other jurisdictions, saw substantial increases in all-cause and overdose mortality.^29,57^ There is a desperate need for studies that explore the mechanisms underlying this crisis.

## Conclusions

To our knowledge, this study provides the most robust evidence to date on the excess risk of COVID-19 mortality among PEH with COVID-19 infection compared with the general population, accounting for population distribution differences by sex and race and ethnicity between the general population and PEH. Excess risk of mortality for PEH vs the general population was observed across all age groups, among male individuals and female individuals, and among Black, Hispanic, and White subpopulations. These excess risks persist despite the likely undercounting of fatalities among PEH and the extensive efforts to protect thousands of the most vulnerable PEH by isolating them in hotels and other short-term housing. Our estimates are consistent with an acceleration of age-related COVID-19 fatality risk of 10 years for PEH in comparison with the general population and a 15-year acceleration of risk compared with White members of the general population. Our results add weight to the notion of homelessness as an accelerant to senescent decline and as a syndromic medical condition that acts as a comorbidity to a wide range of diseases. Our findings also add to the increasing body of evidence that COVID-19 is a housing-sensitive condition and that intersectional identities affect PEH health risks in complex ways. More aggressive housing and homelessness prevention interventions are needed to mitigate the consequences of COVID-19 and other housing-sensitive conditions.

## Notes

Supplement.
Data Sharing Statement
