# Supplementary material for: Race and Ethnicity and Sex Variation in COVID-19 Mortality Risks Among Adults Experiencing Homelessness in Los Angeles County, California
Source: JAMA Netw Open. 2022 Dec 6;5(12):e2245263. doi: 10.1001/jamanetworkopen.2022.45263 (PMC9856229; doi:10.1001/jamanetworkopen.2022.45263)
Supplement: Supplement. — Data Sharing Statement [file jamanetwopen-e2245263-s001.pdf]

Data Sharing Statement

Porter. Race and Ethnicity and Sex Variation in COVID-19 Mortality Risks Among Adults Experiencing Homelessness in Los Angeles County, California. *JAMA Netw Open*. Published December 06, 2022. doi:10.1001/jamanetworkopen.2022.45263

Data
Data available: No

Additional Information
Explanation for why data not available: The data comes from the LA Department of Public Health which restricts the use of de-identified data.
